# The Prevalence of Narcissistic Vulnerability in Men in English Prisons After Criminal Conviction for Stalking

**DOI:** 10.1002/cbm.2388

**Published:** 2025-05-20

**Authors:** Gemma Dearn, Jennifer Bradbury, Helen Thomas, Rachael Wheatley

**Affiliations:** ^1^ His Majesty's Prison Service London UK; ^2^ School of Psychology University of Derby Derby UK

**Keywords:** attachment style, narcissistic vulnerability, stalkers, stalking, treatment needs

## Abstract

**Background:**

In earlier research with prisoners, we observed that convicted stalkers had skill deficits in interpreting their experiences of stalking and their motivations for it, suggesting narcissistic vulnerability.

**Aims:**

Our primary aim was to explore the prevalence of narcissistic vulnerability in men serving a prison sentence in England and to investigate differences in narcissistic vulnerability and attachment styles between men convicted of stalking offences and men convicted of other offences but serving similar sentences.

**Methods:**

Participants were from across 16 closed custodial settings in England. Everyone serving a sentence for a stalking offence was invited to participate together with a same size sample of men serving similar sentences for other offences and without a stalking history. 25%–30% of the eligible men agreed to participate. Each completed three psychometric scales, rating themselves on the Narcissistic Vulnerability Scale (NVS), the Brief‐Pathological Narcissism Inventory (B‐PNI) and the Experiences in Close Relationships‐Revised (ECR‐R) scale. A series of independent sample *t*‐tests were used to compare the experimental group to the other‐conviction control group.

**Results:**

Twenty‐nine individuals sentenced for stalking offences and 25 other prisoners, all men, completed. The stalking group had significantly higher mean scores on narcissistic vulnerability according to both scales and significantly higher mean attachment style difficulties together with higher mean anxiety scores and avoidant scores.

**Conclusions:**

Our findings add data on aspects of personality to a limited pool that supports understanding of men convicted of stalking. Although our sampling and data collection were both limited by the COVID‐19 pandemic conditions, our findings further evidence the case for intervention with respect to ameliorating the personality characteristics of narcissistic vulnerability and attachment styles of such men.

## 1 Introduction

Stalking is a social—and criminal—problem, characterised by targeted, unwanted, repeated patterns of often complex behaviours that can cause fear or distress (Kropp et al. 2011; Mullen et al. 2009; Sheridan et al. 2016). Given the complexities of the behaviours, the definition is not entirely straightforward. For England and Wales, the Stalking Protection Act (2019) gives police powers to apply to a magistrate's (lower) court for a stalking prevention order, and, thus, one definition is simply being in breach of that. In some ways, however, this Act has a complicated definition because the burden of proof required for a prevention order and a criminal conviction differs (see Section 2A and Section 4A). Further, the term stalking is generally underused in crime recording (see HMICFRS, n.d.). The general public, while recognising the concept, also struggle to understand its meaning (Sheridan et al. 2001).

One in five women and one in 10 men will become victims of stalking at some point in their lives (ONS 2020). Although certain stalking‐related behaviours, such as leaving gifts and sending pleasant communication, can appear harmless in isolation, stalking can cause not only devastating primary victim impacts, including physical and/or psychological harms, but also secondary harms, including loss of work and/or breakdown of relationships (ScotCen Social Research 2022). Such impacts are so pervasive and far‐reaching that even children of survivors of stalking have exhibited symptoms of post‐traumatic stress disorder (PTSD; Elklit et al. 2019).

People who stalk are understood to have specific and complex needs (HMICFRS and HMCPSI 2017), yet despite emerging knowledge of risk factors associated with stalking (T. E. McEwan et al. 2017), significant limitations remain regarding understanding stalking desistance (HMICFRS and HMCPSI 2017). In practice, motives, risks, underlying functions and psychopathology of individuals who stalk are not homogenous (MacKenzie et al. 2009; Mullen et al. 1999; Wheatley et al. 2020a), and there is no unifying explanatory model (Meloy and Fisher 2005; Leigh and Davies 2021; Purcell and McEwan 2018). Fundamentally, it is accepted that stalking is a behavioural response to another person given underlying psychological vulnerability, but more research is needed into ‘clinically meaningful constructs that can guide the assessment, formulation and treatment of this client group’ (Parkhill et al. 2022, 562). In addition, current evidence for the efficacy of psychological treatments for stalkers is very limited; therefore, a ‘multifaceted, formulation‐based approach is likely to be required’ (Leigh and Davies 2021, 48).

In attempting to categorise the different subgroups of people who stalk, Mullen et al. (1999) proposed five typologies: *intimacy seeker*, *incompetent suitor*, *rejected*, *resentful* and *predatory,* each emphasising the context in which the stalking arose and initial motives for it. Given that up to half of stalkers return to such behaviours, notably towards their original victim (MacKenzie et al. 2009; T. E. McEwan et al. 2017), and there are no empirically tested and psychologically informed treatment options available for stalking per se, the facilitation of further research in this field is vital (Purcell and McEwan 2018). This, in turn, makes it difficult for risk assessors and treatment providers to plan effective risk management for these individuals (T. E. McEwan et al. 2017) and judge which perpetrators and victims have the greatest need for services and protection (Kropp et al. 2011). Recurrent features of men convicted of stalking include negative perceptions of self but positive perceptions of others, combined with an emotional dependence on gaining acceptance and approval from others, including real (or imagined) intimate partners (Marazziti et al. 2015; Wheatley et al. 2020b). Narcissism has therefore been a significant consideration within this research. Narcissism is generally considered to have two main components: grandiosity, characterised by overt expressions of feelings of superiority and entitlement, and vulnerability, which reflects hypersensitivities and introversive absorbedness (Jauk et al. 2017). Although both constructs share the central concept of self‐centredness (e.g., Brown et al. 2016), they manifest phenomenologically in very different personality types, which Wink (1991) originally referred to as the ‘two faces’ of narcissism. Grandiose narcissists are extraverted, socially bold and even charming (Back et al. 2010; Dufner et al. 2013; Jauk et al. 2016), whereas vulnerable narcissists are introverted, defensive and avoidant (Miller et al. 2011; Hart et al. 2017). Clinical evidence is suggestive of vulnerable narcissism always accompanying grandiose narcissism (Kernberg 1975), but, to date, little is known about the transition between these states (Pincus and Lukowitsky 2010). Further research has outlined the relationship between vulnerable narcissism and a higher tendency towards anger and hostility (Czama et al. 2019), common behaviours seen among stalkers.

Narcissistic vulnerability is defined by the constant need for validation from another, which stems from a deficit of self‐worth (Cain et al. 2008). People who stalk are thought to have predisposing deficits in forming and maintaining healthy intimate relationships (Lewis et al. 2001); indeed, a considerable body of evidence exists supporting links between stalking and the presence of an insecure attachment style (see Parkhill et al. 2022; Dutton et al. 1994; MacKenzie et al. 2008; Wheatley et al. 2020a). Stalking has been labelled as a manifestation of an ‘extreme attachment disorder’ (Meloy 1996, 37), impacting emotion regulation ability. It has also been suggested that ‘obsessional following’—a precursor to the term ‘stalking’—appeared to be a maladaptive response to social incompetence, social isolation and loneliness. A small study of prisoners in Italy, with important links to this current study, concluded that those who stalked were motivated by rejection perception, impulsivity, attachment‐other idealisation, separation anxiety and an egocentric belief in being right (Civilotti et al. 2020). Consistent with this knowledge around treatment pathways, wherein some literature notes that early life experiences are central to the development of narcissistic vulnerability, is a suggestion of the need to explore negative fantasies and their links to feelings of rejection and conflict in relationships within treatment (Busch et al. 2016). It remains to be seen, however, whether individuals convicted of stalking‐related offences, relative to those with non‐stalking‐related offences, differ on psychometric indices of narcissistic vulnerability.

In the study presented here, our aim was a quantitative comparison of the presence of narcissistic traits and insecure attachment styles between men in closed custodial settings in the United Kingdom, one group of whom had one or more criminal convictions for stalking or harassment and one group who had convictions for offences of similar seriousness but not including stalking. Specifically, we set out to test a number of hypotheses:

**Hypothesis One** **(** H1 **)**: *The level of narcissistic vulnerability, as measured by the Narcissistic Vulnerability Scale (NVS), will be higher amongst the stalking group in comparison to the nonstalker but criminal group.*

**Hypothesis Two** **(** **H2** **)**: *The level of narcissistic vulnerability, as measured by the Brief‐Pathological Narcissism Inventory (B‐PNI), will be higher amongst the stalking group in comparison to the nonstalker but criminal group.*

**Hypothesis Three** **(** **H3** **)**: *On the B‐PNI, the stalking group will have higher scores on the subscales of ‘contingent self‐esteem’, ‘hiding the self’ and ‘devaluing’ than the nonstalking criminal group.*

**Hypothesis Four (** **H4** **)**: *The level of insecure attachment style, tested by the Experiences in Close Relationships‐Revised (ECR‐R) scale, will be higher amongst the stalking group than in the nonstalker but criminal group.*

## 2 Methods

### 2.1 Ethics

Ethical approval was obtained from the HMPPS National Research Committee (Reference 2021‐060) and the data protection team of the custodial organisation wherein participants were recruited.

### 2.2 Participants

This sample was derived from a population of men in custodial settings within the Midlands region of England, an area with both rural and dense urban areas, serving sentences of imprisonment ranging from 42 weeks to *life*. The prison settings included both local remand and longer‐term establishments; all were closed prisons with security categorisation levels of B or C (A is the highest). No accessed prisons had a therapeutic community provision, but at least one had a psychologically informed planned environment (PIPE). Participants were initially identified through the core recording systems using offence type as a search criterion and cross‐checked using the Offender Assessment System (OASys) to ensure all participant suitability. Because of the low numbers of individuals in custody convicted of stalking and/or harassment, these offences were grouped together; thus, a conviction of stalking and/or harassment was considered as matching the inclusion criteria. Ninety‐nine men convicted of stalking and/or harassment offences were identified. One hundred age and sentence length‐matched men with no stalking and/or harassment behaviours on their record were selected as the comparison group.

### 2.3 Further Procedures

Each of the selected men was invited to take part in the research via the internal postal system. Recruitment was started in August 2021, so there were restrictions on face‐to‐face contact because of COVID‐19. Although prospective participants could ask questions about the research before deciding on participation, restrictions meant that this generally did not happen but that the men either signed a consent form and handed it to a member of the prison psychology staff or did not proceed further. Consenting participants were then provided with the questionnaires (described below) and with instructions on how to complete and return these. Participants were then provided with a debrief summary reiterating the aims and hypotheses of the research and outlining contact details of supportive services (including Samaritans, a charity providing support to anyone in emotional distress and/or at risk of suicide), information around how to withdraw from the research and how to contact the lead researcher.

### 2.4 The Questionnaires

#### 2.4.1 The Narcissistic Vulnerability Scale (NVS; Crowe et al. 2018)

The NVS is an 11‐item measure of ‘vulnerable narcissism’ (e.g., ‘In general, I feel self‐absorbed’) measured using a 5‐point self‐report scale (from ‘*Not at all*’ to ‘*Extremely*’). The NVS is the first validated measure of vulnerable narcissism, with the capacity for measuring short‐term fluctuations in vulnerable narcissism (Crowe et al. 2018). The ICC (intraclass correlation coefficient) was excellent, indicating a high degree of agreement among experts on the items most characteristic of narcissistic vulnerability (Hallgren 2012), and factor analyses revealed that the NVS is an internally consistent, unidimensional measure at both the between‐ and within‐person levels. Specific cut‐off scores for the NVS are not universally established; the interpretation of scores can vary depending on the context and the population being studied. Typically, higher scores on the NVS indicate greater levels of narcissistic vulnerability; it is, however, important to have a qualified professional interpret these scores within the broader clinical context. In this study, scores were compared between the two groups and mean differences tested for significance, but normative data are not well established, so clinical interpretation is not established.

#### 2.4.2 The Brief‐Pathological Narcissism Inventory (B‐PNI; Schoenleber et al. 2015)

The B‐PNI is a 28‐item measure, developed as a ‘brief’ but comprehensive measure of pathological narcissism encompassing the concept of narcissistic grandiosity as well as vulnerability. Narcissistic grandiosity is measured across the facets of entitlement rage, exploitativeness, grandiose fantasy and self‐sacrificing self‐enhancement (e.g., ‘I feel important when others rely on me’.), whereas narcissistic vulnerability is measured across the facets of contingent self‐esteem, hiding the self and devaluing (e.g., ‘When others don't meet my expectations, I often feel ashamed about what I wanted’.). Each item is scored using a 5‐point self‐report scale (from ‘*Not at all like me*’ to ‘*Very much like me*’). Original scale analyses (Schoenleber et al. 2015) supported the criterion validity and utility. The B‐PNI consists of seven subscales, each of which requires a total calculated score. Specific cut‐off scores are not universally established for the B‐PNI and can vary depending on the population being studied and the context of the assessment. Typically, higher scores indicate greater levels of pathological narcissism; the interpretation of these scores should be done by a qualified professional who can consider the broader clinical picture. For this study, scores were compared between groups, but no population‐based norms have been established.

#### 2.4.3 The Experiences in Close Relationships‐Revised (ECR‐R; R. C. Fraley et al. 2000)

The ECR‐R is a 36‐item measure of adult attachment style across the facets of avoidance (e.g., ‘I tell my partner just about everything’.) and anxiety (e.g., ‘I worry a lot about my relationships’.). Each item is scored using a 7‐point self‐report scale (from ‘*Strongly Disagree*’ to ‘*Strongly Agree*’). It provides a reliable and replicable dual‐factor self‐report measure of adult romantic attachment. Results suggest that the ‘scale maintains acceptable classical psychometric properties while also assessing a range of trait scores more evenly distributed than previous measures’ (R. C. Fraley et al. 2000). The cut‐off scores for the ECR‐R are not universally fixed; however, researchers often use thresholds to categorise attachment styles based on the score ranges for the anxiety and avoidance dimensions. For example, a score of between 4 and 7 on both the anxiety and avoidance subscales would indicate ‘high anxiety and high avoidance’ (a fearful‐avoidant attachment style). Cut‐off scores are, however, approximations based on common interpretation in the literature. No cutoff was used to clinically interpret the group scores in this study.

### 2.5 Analytic Plan

Data were analysed using SPSS v.28. A post hoc power analysis indicated good statistical power (0.9) despite the small sample size. After establishing independence, normality of distribution and homogeneity of variance, independent samples *t*‐tests were used to compare mean scores on the composite and subfacet scores of the narcissism and attachment measures between experimental and control groups.

## 3 Results

### 3.1 General Description of the Sample

In total, 29 (29%) of the 99 men identified for the stalking group participated. One had to be excluded on grounds of not speaking/reading sufficient English, and one because his mental state was regarded as too fragile. The remainder of the nonparticipants simply did not respond to the invitation to participate. Just 25 of the 100 eligible men with other convictions participated.

Ages in the stalker group ranged from 20 to 61 years (*M* = 37.48, SD = 10.14); the nonstalker comparison men were significantly younger (*M* = 31.12, SD = 10.27 [range 20–61]; *t*(52) = 2.19, *p* < 0.05). Twenty‐three of the stalker group and 19 of the nonstalkers were of white British ethnicity. Sentence length within both groups ranged between 42 weeks and life; two participants in the stalker group and five in the nonstalker group were serving life sentences. Four participants in the stalker group and three in the nonstalker group were unsentenced. When removing these extremes (life sentence or unsentenced), the average sentence lengths for the stalker group (*M* = 52.8, SD = 37.7) and the nonstalker group (*M* = 33.9, SD = 30.1) were not significantly different (*t*(38) = 1.74, *p* > 0.05).

### 3.2 Preliminary Narcissism Hypothesis Testing

The results of all analyses are shown in Table 1.

**TABLE 1:** Comparison of narcissism and Experience in Close Relationships‐Revised scale scores between men imprisoned for a stalking offence and men serving a similar sentence for any other offence.

		*M*	SD	*t*	df	*p*	Cohen's *d*
NVS	Stalking conviction	42.19	16.3	2.63	43.56	0.006	0.76
Total	Control	30.50	13.8				
B‐PNI	Stalking conviction	56.55	35.1	3.69	45.15	< 0.001	0.97
Total	Control	28.40	19.7				
B‐PNI	Stalking conviction	7.17	6.6	3.50	41.76	< 0.001	0.19
Contingent self‐esteem	Control	2.32	3.2				
B‐PNI	Stalking conviction	4.86	4.6	0.77	48.6	0.44	0.20
Exploitative	Control	4.04	3.0				
B‐PNI	Stalking conviction	10.9	6.3	0.94	50.58	0.35	0.25
Self‐sacrificing	Control	9.33	5.7				
B‐PNI	Stalking conviction	11.0	6.3	3.22	48.87	0.002	0.85
Hiding the self	Control	6.32	4.2				
B‐PNI	Stalking conviction	8.86	6.5	2.04	51.85	0.04	0.55
Grandiose	Control	5.56	5.3				
B‐PNI	Stalking conviction	6.79	6.2	2.44	44.06	0.018	0.64
Devaluing	Control	3.52	3.3				
B‐PNI	Stalking conviction	6.97	6.1	1.34	51.88	0.186	0.36
Entitlement rage	Control	4.92	5.0				
ECR‐R	Stalking conviction	3.53	1.44	2.96	45.17	0.002	0.79
Anxiety	Control	2.56	0.86				
ECR‐R	Stalking conviction	3.97	0.10	2.51	34.92	0.008	0.74
Avoidant	Control	3.86	0.17				

Abbreviations: B‐PNI, Brief‐Pathological Narcissism Inventory; ECR‐R, Experiences in Close Relationships‐Revised; NVS, Narcissistic Vulnerability Scale.

H 1 An independent t‐test indicated that the group convicted of stalking had a significantly higher mean score on the Narcissistic Vulnerability Scale than the nonstalker group (t(2.63) = 43.56, *p =* *0.006*), with a medium effect size.

H 2 An independent t‐test confirmed that the group convicted of stalking offences had significantly higher mean total scores on the Brief‐Pathological Narcissism Inventory (B‐PNI) than the nonstalker group (t(3.69) = 45.15, p = 0.001), with a large effect size.

H 3 All three subscales of the Brief‐Pathological Narcissism Inventory (B‐PNI) hypothesised as specifically distinguishing between the groups of men—‘contingent self‐esteem’, ‘hiding the self’ and ‘devaluing’—did so, with higher mean scores in the stalking group, whereas the other subscales—‘exploitative’, ‘self‐sacrificing self‐enhancement’, ‘Grandiose fantasy’ and ‘entitlement rage’—did not.

Contingent self‐esteem—t(3.50) = 41.77, p = 0.001, with a large effect size.

Hiding the self—t(3.22) = 48.88, p = 0.002, with a large effect size.

Devaluing—t(2.45) = 44.06, p = 0.018, with a medium effect size.

H 4 Mean scores on the ‘attachment‐related anxiety’ scale were higher in the stalking group than the other offenders (t(2.97) = 45.17, p = 0.002), with a medium effect size, and on the ‘attachment‐related avoidance’ scale (t(2.52) = 34.92, p = 0.008), again with a medium effect size.

## 4 Discussion

In line with our hypotheses, the group of men with convictions for stalking‐related offences recorded significantly higher mean scores on psychometric measures of composite narcissistic vulnerability, as well as its subcomponents of contingent self‐esteem, hiding the self and devaluing, and also showed higher mean self‐ratings on avoidant and anxious attachment styles than those convicted of non‐stalking‐related offences. These findings are in line with other recently published studies. Wheatley et al. (2020b), for example, found narcissistic vulnerability in men convicted of stalking, perhaps driven by underlying psychosocial vulnerabilities. Our findings also support previous posits about the links between stalking and narcissism (Meloy 1999) and the presence of some form of psychological vulnerability underlying stalking consistently appearing in existing stalking theories (e.g., Parkhill et al. 2022).

Our findings also support previous literature suggesting that underlying psychosocial vulnerabilities drive the need for validation from another person (Marshall 1989) and of pointers towards suggestions that stalking, in part, allows the stalker to salvage their damaged self‐esteem and, in turn, feel better about themselves (MacKenzie et al. 2008). Contingent self‐esteem is considered to be fragile, fluctuating and only maintained when the individual is able to successfully meet the standards upon which his or her self‐esteem is based (Zeigler‐Hill et al. 2008). Thus, individuals identified as vulnerable narcissists are believed to rely primarily upon the approval of others (Cooper and Maxwell 1995).

The notion of ‘hiding the self’ is characterised by dependency and feeling weak and shameful and is further highlighted through individuals being likely to conceal their needs, concerns and imperfections (Naidu et al. 2019). Again, our findings support previous research (Wheatley et al. 2020a), wherein men convicted of stalking reported engaging in such behaviours partially due to experiencing insecurities and loneliness, which, in turn, influenced levels of self‐esteem. Such deficits resulted in stalking behaviours, whereby the victims were the only sense of true meaningful connection. ‘Devaluing’ is characterised as individuals showing ‘disinterest in and avoidance of others who do not provide needed admiration’ (Naidu et al. 2019). Findings of this research therefore support Marazziti et al. (2015), who suggested that those who stalk are emotionally dependent on attempts to gain acceptance and approval from desired others. Taken together, our findings suggest that higher levels of contingent self‐esteem, devaluation of others and hiding the true self from others might drive risk for stalking behaviours and, as such, should be considered for inclusion within both treatment planning and risk assessing practices and client engagement.

Further analyses pertaining to attachment differences were indexed through differences across anxious and avoidant subfacets of the ECR‐R. Although those with an anxious attachment style tend to have low self‐esteem and a negative view of themselves, individuals with avoidant attachment styles often view themselves as being significantly independent and self‐sufficient (C. R. Fraley et al. 2011). Findings of this research indicated support for increased attachment‐related anxiety and avoidance in those with convictions for stalking relative to other‐conviction controls, thus supporting extant links between emotional loneliness, intimacy deficits and offending behaviour (Marshall 1989). Overall, insecure attachments, and more specifically anxious attachment styles, result in an individual being hypersensitive to threat, exhibiting coercive and controlling behaviours (Ainsworth and Bowlby 1991; Dutton et al. 1994). This might account for some of the offence‐related behaviours enacted by those convicted of stalking and, as such, provide a potential practical avenue for early prevention and/or treatment targets.

Our study thus adds evidence to the currently limited empirical base for understanding and, ultimately, managing stalking in terms of psychological vulnerabilities. It is vital now to test interventions to improve mechanisms for the capacity to manage the sense of self‐worth without intruding on others' safety and sense of security.

### 4.1 Limitations

The main limitation of our study was the fact that we had to complete it during the COVID‐19 pandemic and thus had no personal contact with potential participants—which may have affected both the level of recruitment and responding styles. Over two‐thirds of eligible prisoners chose not to participate, although the response rate was similar between the groups. It is possible, therefore, that not only was the sample of 59 underpowered for the tasks, but also unrepresentative. We have already referred to post hoc power checks, but the main concern here is that there may have been additional features distinguishing the stalkers that we could not identify. Further, we were unable to consider subgroups of stalkers—and stalking and harassment offences can have very different motivations/functions; combining all types as we had to do may be misrepresentative of one or both groups. Further, no data regarding the participants' relationship with victim(s) were gathered; it could be helpful to revisit the prevalence of traits explored in this study, focusing on any differences between stalking typology and relationship with the victim(s) to ascertain whether a narcissistic vulnerability is more apparent in a particular group.

Informal feedback from participants who declined to take part in the research indicated that those convicted of stalking did not wish to associate themselves with the ‘stalking’ or ‘stalker’ labels in so much that they refuted their convictions and thus did not consider themselves to meet the research criteria. This aligns with the findings of Wheatley et al. (2020a), whereby individuals had a strong desire to distance themselves from such labels; this information should be used to inform future research in this field. Further, owing to the COVID‐19 pandemic‐related restrictions in place, data had to be collected remotely, so although we believe that the men included in this study understood the research as a whole and how to complete the questionnaires, the research team was not on hand to monitor the participants when completing the scales—to ensure that the man recruited was in fact the man completing the questionnaire or to ensure a thorough understanding of each item and the timely and accurate completion of the questionnaire pack. It would be interesting to replicate the study with researchers present. Third, although statistically significant differences were identified between experimental and control groups across all metrics, clinical interpretations were not possible and specific understanding as to the precise impact these variables have on stalking behaviour remains unclear. As such, future programmes of research would benefit from life‐course developmental and/or reflective‐qualitative work to further delineate this linkage.

### 4.2 Practice and Research Implications

This research supports the hypothesis that men convicted of stalking offences are more likely than other prisoners to have higher levels of narcissistic vulnerability and attachment problems, adding weight to prior research. Findings suggest advantages in including such measures for risk assessment for such men (also see, e.g., risk factors related to ‘entitlement’ in MacKenzie et al. 2009) and for treatment (see T. McEwan et al.'s (2024) discussions about ‘entitlement’ in treatment). Similarly, treatment development might thus be designed to (1) explore negative fantasies held about oneself, (2) make connections between fantasy and sensitivity to rejection and disappointment to aid in recognition of distorted perceptions about others and one’s own value and (3) examine defensive responses to vulnerability, which, in turn, perpetuate conflict, frustration and disappointment in relationships (Busch et al. 2016) and to factor these into personalised treatment. Additional qualitative research could be beneficial to help interpret and qualify the findings reported here. Specifically, the exploration of self‐awareness of narcissistic vulnerability and the direct impact this has on stalking as an offending behaviour, as well as reflections on attachment styles, their manifestation and any associated perceived impact they have on their stalking behaviours.

## 5 Conclusion

This research directly addresses gaps in understanding those convicted of serious enough stalking behaviours that they have been sentenced to imprisonment. It adds weight to prior research‐informed indications of the role of the narcissistic vulnerability personality trait as predisposing to, precipitating and/or perpetuating stalking‐related behaviours. It could help guide the development of treatment planning and risk assessment of this convicted group.

## Ethics Statement

Research ethics approval was obtained from the National Research Committee.

## Consent

The authors have nothing to report.

## Conflicts of Interest

The authors declare no conflicts of interest.

## Data Availability

The data that support the findings of this study are available upon request from the corresponding author. The data are not publicly available due to privacy or ethical restrictions.
